# Effects of Streptozotocin-Induced Diabetes on the Pineal Gland in the Domestic Pig

**DOI:** 10.3390/ijms19103077

**Published:** 2018-10-09

**Authors:** Bogdan Lewczuk, Magdalena Prusik, Natalia Ziółkowska, Michał Dąbrowski, Kamila Martniuk, Maria Hanuszewska, Łukasz Zielonka

**Affiliations:** 1 Department of Histology and Embryology, Faculty of Veterinary Medicine, University of Warmia and Mazury in Olsztyn, Oczapowskiego 13, 10-719 Olsztyn, Poland; mprusik@gmail.com (M.P.); ntrzaska@o2.pl (N.Z.); kamila.kwiecinska91@gmail.com (K.M.); marysia-h@wp.pl (M.H.); 2 Department of Veterinary Prevention and Feed Hygiene, Faculty of Veterinary Medicine, University of Warmia and Mazury in Olsztyn, Oczapowskiego 13, 10-719 Olsztyn, Poland; michal.dabrowski@uwm.edu.pl (M.D.); lukaszz@uwm.edu.pl (Ł.Z.)

**Keywords:** diabetes, pineal gland, melatonin, serotonin, norepinephrine, indoles, catecholamines, streptozotocin, pig

## Abstract

Several observations from experiments in rodents and human patients suggest that diabetes affects pineal gland function, including melatonin secretion; however, the accumulated data are not consistent. The aim of the present study was to determine the effects of streptozotocin-induced diabetes on the pineal gland in the domestic pig, a species widely used as a model in various biomedical studies. The study was performed on 10 juvenile pigs, which were divided into two groups: control and diabetic. Diabetes was evoked by administration of streptozotocin (150 mg/kg of body weight). After six weeks, the animals were euthanized between 12.00 and 14.00, and the pineal glands were removed and divided into two equal parts, which were used for biochemical analyses and for preparation of explants for the superfusion culture. The pineal contents (per 100 μg protein) of serotonin, 5-hydroxyindole acetic acid, 5-hydroxytryptophol, 5-methoxyindole acetic acid, 5-methoxytryptophol, and 5-methoxytryptamine were significantly lower in diabetic pigs than in control pigs. In contrast, the level of N-acetylserotonin was significantly higher in diabetic animals. No significant differences were found in the level of melatonin between control and experimental pigs. The amounts of 3,4-dihydroxyphenylalanine, dopamine, norepinephrine, and 3,4-dihydroxyphenylacetic acid were significantly lower in the pineal glands of diabetic animals. The level of vanillylmandelic acid was higher in diabetic pigs. No differences were observed in the level of basal and NE-stimulated release of N-acetylserotonin or melatonin between the pineal explants prepared from control and experimental animals. In vitro treatment with insulin was ineffective. In conclusion, streptozotocin-induced diabetes affects both indole metabolism and adrenergic neurotransmission in the pig pineal gland.

## 1. Introduction

There is growing evidence of interplay between the pineal gland and the pancreatic islets. The activities of both endocrine structures change markedly with the diurnal rhythm, ensuring synchronization of various physiological processes with daily fluctuations in environment [1,2,3]. Secretion of melatonin is elevated at night in almost all studied species [1], whereas the course of insulin secretion rhythm, according to the current state of knowledge, differs between diurnal and nocturnal species [2,4,5]. In humans, the insulin secretion rhythm is 180° out of phase with the melatonin rhythm, while in nocturnal rodents, including the rat, the peak of insulin is concomitant with or precedes that of melatonin [2,4,5]. Experiments in superfusion culture have revealed that isolated rat pancreatic islets secrete insulin in an autonomously generated circadian rhythm [6,7,8]. Further studies provided data about the molecular organization of circadian oscillator in the pancreatic islets [9,10,11,12]. Melatonin acts as a synchronizing agent (zeitgeber) on the islets’ circadian oscillator, inducing a shift in the rhythm of insulin secretion [5,6,12,13]. The mechanisms of melatonin action on β-cells differ between animals [5,12,13,14]. In the rat, melatonin negatively affects insulin secretion both in vitro and in vivo [5,6,15]. Pinealectomy was found to increase average glucose levels in rodents [16]. Contrary to data from experiments in the rat, melatonin evoked an increase in insulin release from human pancreatic islets in vitro [17].

Several observations from rodent models and human diabetic patients suggest that important feedback mechanisms exist between the pancreatic islets and the pineal gland [18]. The pineal gland and its melatonin-synthesizing machinery are sensitive to changes in insulin and glucose levels; however, the reported results are not consistent [18,19,20,21,22,23]. Reduced levels of pineal or/and blood melatonin were described in alloxan- and streptozotocin-induced diabetic rodents [23,24,25], type 2 diabetic rats [26,27,28], and in human patients with diabetes [28,29,30]. However, other reports show increased melatonin synthesis in streptozotocin-induced diabetic rats [19] and in rats with mutation-induced diabetes type 1 [31]. In rat pineal gland cultures, Garcia et al. [22] showed that insulin potentiates the effect of norepinephrine (NE) on melatonin synthesis. It should be noted that insulin receptors are expressed in pinealocytes [32]. There are no published data on the effect of diabetes on pineal gland functions in non-rodent animal species. 

The present study investigates the effect of streptozotocin-induced diabetes on the pineal gland of the domestic pig. This species is widely used as a model for human physiology and pathology because the structural and functional organization of many organs of the pig resemble those of the human [33]. Especially in the field of metabolic disorders, including diabetes, the pig is a highly promising model [34,35]. Many processes occurring at the molecular, cellular, tissue, and organ levels during diabetes mimic that observed in humans. The analysis and interpretation of data obtained in biomedical experiments performed on pigs require the detailed knowledge of the physiology and pathophysiology of this species.

The morphology and physiology of the pig pineal gland show several species-specific features concerning mechanisms of adrenergic regulation of melatonin secretion [36,37], daily patterns of plasma melatonin level [38,39,40], and the ultrastructure of pinealocytes [41,42,43]. In contrast to the rat pineal gland, norepinephrine stimulates melatonin synthesis in pig pinealocytes without an induction of transcription and translation of serotonin N-acetyltransferase [36,37], most likely by an increase in the affinity of this enzyme to serotonin [44]. Transcription-dependent mechanisms are proposed to be involved in long-term regulation of melatonin synthesis [45]. Specific regulation of secretory activity seems to be responsible for the characteristic patterns of diurnal changes in circulating melatonin levels [38,39,40,46,47,48]. 

In view of the facts that (i) the current knowledge on the influence of diabetes on the pineal gland is almost exclusively the result of experiments performed in the rat; (ii) the pig is an important model in biomedical studies on metabolic disorders; and (iii) there are prominent inter-species differences in the pineal physiology, the aim of study is to determine the effects of streptozotocin-induced diabetes on pineal activity in the domestic pig.

## 2. Results

### 2.1. In Vivo Study

#### 2.1.1. Content of Melatonin Synthesis-Related Indoles

**Figure 1:**
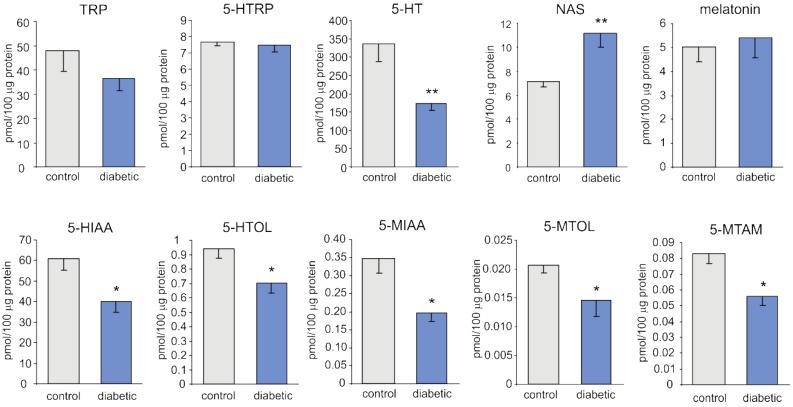
The content (per 100 μg of protein) of melatonin synthesis-related indoles in the pineal glands of control and diabetic pigs. The means significantly different from those of the control group are marked with asterisks; single asterisk (*) indicates *p* < 0.05, double asterisk (**) represents *p* < 0.01. Abbreviations: TRP, tryptophan; 5-HTRP, 5-hydroxytryptophan; 5-HT, serotonin; NAS, N-acetylserotonin; 5-HIAA, 5-hydroxyindole acetic acid; 5-HTOL, 5-hydroxytryptophol; 5-MIAA, 5-methoxyindole acetic acid; 5-MTOL, 5-methoxytryptophol; 5-MTAM, 5-methoxytryptamine.

The contents (per 100 μg of protein) of tryptophan (TRP) and 5-hydroxytryptophan (5-HTRP) did not differ significantly between the pineal glands of control and diabetic pigs (Figure 1). The levels of serotonin (5-HT), 5-hydroxyindole acetic acid (5-HIAA), and 5-hydroxytryptophol (5-HTOL) in the pineal gland were significantly lower in diabetic pigs than in control animals (by 48%, 34%, and 25%, respectively). Similarly, the levels of direct 5-metoxy derivatives of these compounds, 5-methoxytryptamine (5-MTAM), 5-methoxyindole acetic acid (5-MIAA), and 5-methoxytryptophol (5-MTOL), were also markedly lower in streptozotocin-treated animals (by 33%, 43%, and 30%, respectively). In contrast, the content of N-acetylserotonin (NAS) was significantly higher (by 55%) in diabetic pigs than in control pigs. The level of melatonin in the pineal gland did not differ significantly between control and diabetic pigs.

#### 2.1.2. Content of Catecholamines and Their Metabolites

**Figure 2:**
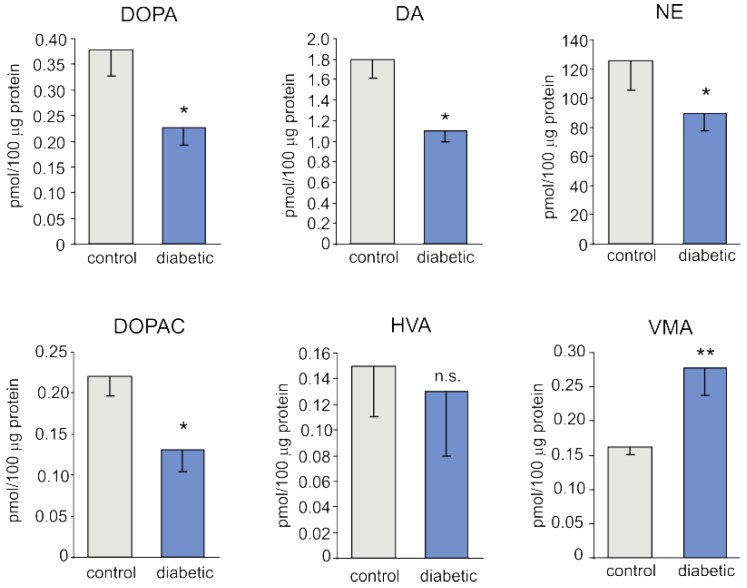
The content (per 100 μg of protein) of catecholamines and their metabolites in the pineal glands of control and diabetic pigs. The values presented are means and standard errors (*n* = 5). The means significantly different from those of the control group are marked with asterisks; single asterisk (*) indicates *p* < 0.05, double asterisk (**) represents *p* < 0.01, n.s. indicates non-significant. Abbreviations: DOPA, 3,4-dihydroxyphenylalanine; DA, dopamine; NE, norepinephrine; DOPAC, 3,4-dihydroxyphenylacetic acid; VMA, vanillylmandelic acid; HVA, homovanillic acid.

The contents (per 100 μg of protein) of norepinephrine (NE), 3,4-dihydroxyphenylalanine (DOPA), dopamine (DA), and 3,4-dihydroxyphenylacetic acid (DOPAC) in the pineal gland were significantly lower in streptozotocin-treated animals than in control animals (Figure 2). On the other hand, the content of vanillylmandelic acid (VMA) was significantly higher in diabetic pigs than in control pigs. No significant differences between the groups of pigs were found in the level of homovanillic acid (HVA).

### 2.2. In Vitro Study 

**Figure 3:**
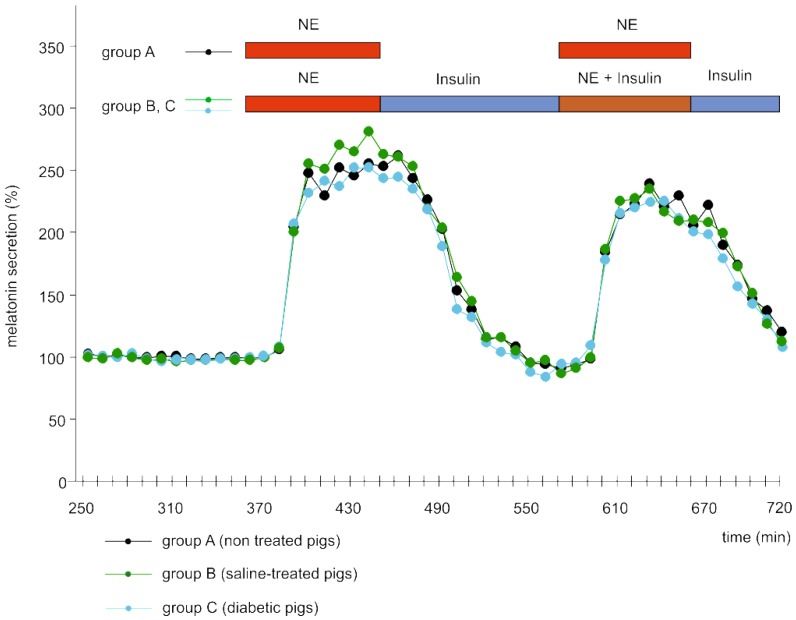
Melatonin secretion from explants of the pineal glands of normal, non-treated pigs (group A), control, saline-treated pigs (from the in vivo experiment, group B) and diabetic, streptozotocin-treated pigs (from the in vivo experiment, group C). The explants from groups A, B, and C were incubated in medium containing 10 μM NE between 361 and 450 min of the experiment. Next, the explants of group B and C were incubated in a medium containing 10 μM of insulin to the end of culture and stimulated with NE (10 μM) between 571 and 660 min of the experiment. The explants of group A were incubated in medium without insulin and treated with NE between 571 and 660 min of the experiment. The mean melatonin secretion between 300 and 360 min of the experiment was taken as 100%. The data presented are means (*n* = 5). Horizontal bars show respective periods of incubation in medium with NE, insulin, or NE + insulin.

**Figure 4:**
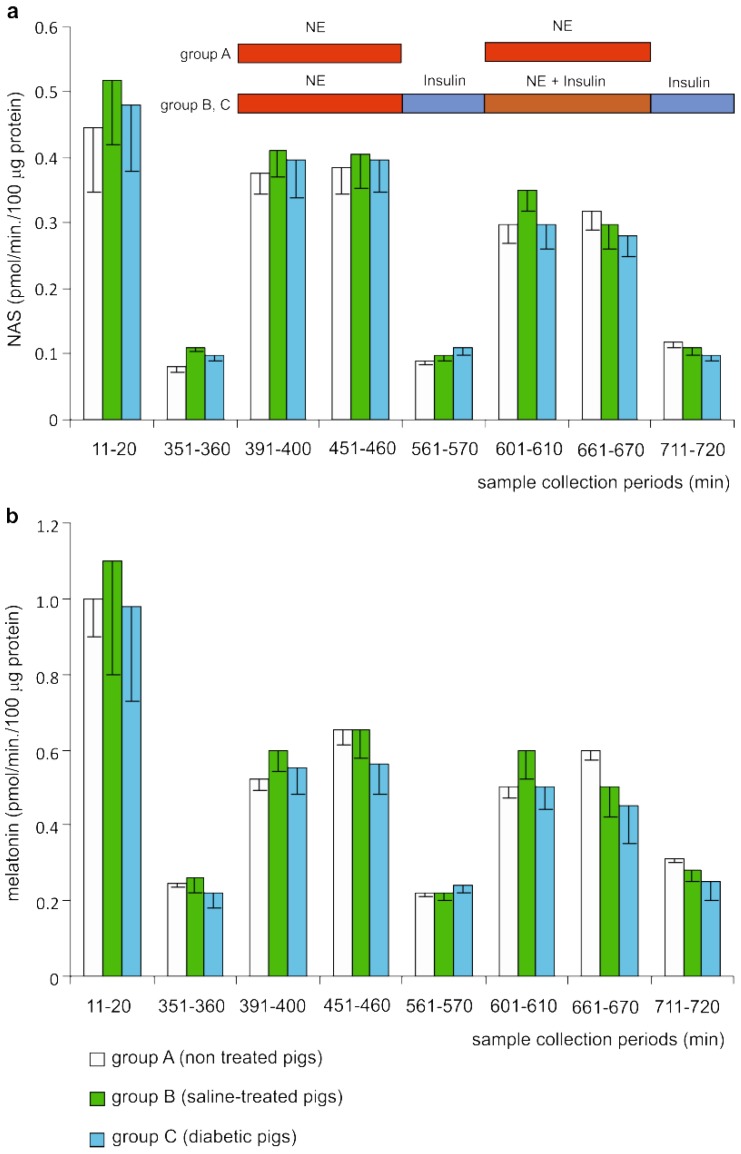
The levels of (**a**) N-acetylserotonin (NAS) and (**b**) melatonin (both measured by HPLC) in selected medium samples collected during the superfusion culture. For details of the experiment, see Figure 3. The values presented are means and standard errors (*n* = 5).

The changes in melatonin secretion from the pineal glands of control and diabetic pigs in response to adrenergic stimulation and insulin treatment were determined by measurement of the hormone concentration using radioimmunoassay (RIA) in all samples collected during the superfusion culture (Section 2.2.1, Figure 3). In view of the fact that the results of our in vivo study showed significant differences in the level of NAS between the pineal glands of diabetic and non-diabetic pigs, concentrations of NAS (and simultaneously melatonin) were determined in selected medium samples by high pressure liquid chromatography technique (HPLC) and presented in Section 2.2.2 (Figure 4).

#### 2.2.1. Melatonin Secretion

Melatonin secretion from pineal explants of untreated, normal pigs (group A) increased rapidly after starting the incubation in a medium containing 10 µM of NE (Figure 3). It reached the maximum in less than 30 min, then remained at a stable, increased level as long as NE at 10 µM was present in the culture medium, and decreased just after withdrawal of the catecholamine from the culture medium. The second stimulation with NE also resulted in a quick increase in melatonin secretion, but the maximal level of hormone release was slightly lower than during the first stimulation. 

There were no significant differences (Figure 3) in the melatonin secretion between the above described group A of explants and the explants obtained from the pineal glands of control pigs treated with saline (group B) and diabetic pig treated with streptozotocin (group C). Norepinephrine induced a similar rise in melatonin secretion from the pineal explants of diabetic (group C) and non-diabetic pigs (groups A, B). Incubation in the medium with 10 μM of insulin before and during the second adrenergic stimulation did not change the levels of basal and NE-induced melatonin secretion from explants of both control (group B) and diabetic pigs (group C), compared to the explants incubated in medium without insulin (group A).

#### 2.2.2. Release of N-acetylserotonin 

The release of NAS (Figure 4) did not differ significantly between explants prepared from the pineal glands of non-diabetic (group A, B) and diabetic pigs (group C). At the beginning of the incubation, the mean release of NAS was approximately 0.45 pmol/min/100 µg of protein in all studied groups, and after 6 h of incubation it decreased to approximately 0.1 pmol/min/100 μg of protein. During the first adrenergic stimulation, the release of NAS increased in groups A, B, and C to 0.376 ± 0.03, 0.411 ± 0.04, and 0.398 ± 0.06 pmol/min/100 μg of protein, respectively. Next, the release of NAS decreased to levels similar to those before the first adrenergic stimulation. The second adrenergic stimulation resulted in a slightly lower increase in the release of NAS than the first stimulation. Treatment with insulin did not change the level of NAS release either before or during the second adrenergic stimulation. The levels of melatonin secretion determined by HPLC are shown in Figure 4.

## 3. Discussion

Our data show that six-week-long streptozotocin-induced diabetes resulted in significant changes in the metabolism of melatonin synthesis-related indoles in the pig pineal gland. The decrease of 5-HT content by almost 50% was one of the most prominent effects of diabetes. The reduced levels of 5-HIAA and 5-HTOL found in diabetic pigs are probably the result of the diminished 5-HT pool because several studies reported a positive correlation between levels of 5-HT and their metabolites forming as a result of oxidative deamination [49]. Similarly, the decrease of 5-MIAA and 5-MTOL contents seem to be caused by reduced levels of 5-HIAA and 5-HTOL. However, at this point, it should be noted that the methylation step, according to a recent study performed in the chicken pineal organ, may play a crucial, rate-limiting role in the formation of 5-MIAA and 5-MTOL in some species [50]. 

Few studies have been published that deal with the effect of diabetes on the profile of pineal indoles, therefore, we have very little data for comparative analysis. Champney et al. [20] showed that the pineal levels of 5-HT, its precursors (TRP, 5-HTRP), and metabolites (5-HIAA) in the rat and the Syrian hamster were not affected by administration of streptozotocin or insulin. Frese et al. [26] reported lower levels of TRP, 5-HTRP, 5-HT, NAS, and melatonin in type 2 diabetic Goto-Kakizaki rats than in Wistar rats. 

When looking for a mechanism leading to the decrease of 5-HT level in the pineal gland of diabetic pigs, the following options should be considered: (i) diminished synthesis of 5-HT; (ii) increased degradation of 5-HT by oxidative deamination; (iii) increased utilization of 5-HT for NAS and melatonin synthesis; and (iv) impaired storage of 5-HT in pinealocytes. The possibility of decreased synthesis of 5-HT in the pineal gland could be excluded because there were no differences in the level of 5-HTRP between control and diabetic pigs. Similarly, analysis of the proportion between 5-HIAA and 5-HT or between 5-HTOL and 5-HT does not provide any support for the idea of increased 5-HT degradation. The level of NAS was significantly higher in diabetic pigs than in the control pigs, which largely supports the idea that increased acetylation of 5-HT is responsible for the decreased levels of this amine. It is worth noting that the pig pineal gland contains extremely high levels of NAS and melatonin, even during photophase. In our opinion, the hypothesis of impaired storage of 5-HT in diabetic pigs should also be considered. The pig pineal gland contains large amounts of this indoleamine, which represent approximately 70% of studied indoles.

The decrease of 5-HT level in the pineal gland as a result of streptozotocin-induced diabetes is of special interest in terms of more general aspects concerning the effect of diabetes on the prevalence of depressive disorders in humans [51], which could be related to changes in serotoninergic brain systems. The decreased content of 5-HT was demonstrated in the brain cortex, hippocampus, striatum, and cerebrospinal fluid of rats with streptozotocin-induced diabetic encephalopathy, compared to control animals [52]. Changes in serotonin metabolism were also reported in streptozotocin-induced diabetic mice [53]. Diabetic animals show dysregulation of serotonergic systems in several brain areas associated with anxiety-like responses [54]. In view of these data, the pineal gland with its high 5-HT level seems to be a promising model for research on the effect of diabetes on brain serotoninergic systems. 

Similar to 5-HT, the content of 5-MTAM was also significantly lower in the pineal glands of diabetic pigs than control animals. Our previous studies showed that species with high levels of 5-HT in the pineal organs have much more 5-MTAM than those with low levels of 5-HT [49,50]. This points to a close relationship between 5-HT and 5-MTAM.

Our results showed that the content of NAS was significantly higher in the pineal glands of diabetic pigs than those of control pigs. The pineal level of melatonin did not differ significantly between groups. Similar to our results, Pang et al. [24] found significantly higher pineal levels of N-acetylserotonin in rats with alloxan-induced diabetes than in control animals. Surprisingly, the pineal and serum levels of melatonin were lower in these diabetic rats than in control rats. Champney et al. [20] failed to demonstrate any effect of streptozotocin-induced diabetes or insulin on the pineal levels of N-acetylserotonin and melatonin as well as activities of serotonin N-acetyltransferase and N-acetylserotonin O-methyltransferase in rats. In contrast, Amaral at al. [23] demonstrated decreased nocturnal secretion of melatonin using a microdialysis method in rats with streptozotocin-induced diabetes. Peschke at al. [19] showed increased plasma melatonin levels in rats with streptozotocin-induced diabetes. In summary, data concerning the effect of diabetes on melatonin levels in vivo, accumulated up till now, are not consistent, even within the same model-streptozotocin-induced diabetic rat. 

In the present work, we studied for the first time the effect of diabetes on biochemical markers of sympathetic nerve terminals in the pineal gland. The level of NE and its precursors, DOPA and DA, were significantly reduced in diabetic pigs compared to control animals. Similarly, the content of DOPAC, a metabolite of DA, was also decreased. On the other hand, the level of VMA, a metabolite of NE, was significantly higher in diabetic pigs than in control pigs. Our results provide strong evidence that streptozotocin-induced diabetes influences sympathetic neurotransmission in the pig pineal gland. Two hypotheses related to these results should be considered. One possibility is that streptozotocin-induced diabetes leads to increased stimulation of sympathetic ganglion cells, the axons of which innervate the pineal gland. The release of NE is augmented for a long time, which is measurable as the increased level of VMA. Increased utilization of NE results in decreased levels of NE and its precursors. At the postsynaptic level, this overstimulation leads to increased levels of NAS (and perhaps melatonin for some period of time) and decreased levels of 5-HT. The second possibility is that diabetes induces pathological changes in the physiology of neurons innervating the pig pineal gland, leading to decreased synthesis and storage of catecholamines. Diabetes-induced neuropathy was observed in intrapineal nerve fibres containing tyrosine hydroxylase in rats with streptozotocin-induced diabetes [55]. The content of NE was lower in the pineal glands of diabetic Goto-Kakizaki rats than in Wistar rats [26]. Further studies are needed to explain the significance and mechanism of changes in the content of catecholamines in the pig pineal gland with diabetes. 

The results obtained in our in vitro experiment clearly show that (i) NE induced similar increases in the release of NAS and melatonin from the pineal glands of normal and diabetic pigs; and (ii) insulin did not change the levels of basal and NE-stimulated release of NAS and melatonin, either from normal or diabetic pigs. The results of our experiment with pig pineal explants differ from those obtained in the rat. Garcia et al. [22] showed that insulin (10^−8^ M) potentiated NE-stimulated melatonin secretion. Looking for the mechanisms of insulin action, the authors found that this hormone augmented NE-induced increases in tryptophan hydroxylase activity and serotonin N-acetyltransferase activity. Amaral et al. [23] reported a decreased release of melatonin during NE-stimulation from the pineal gland of rats with streptozotocin-induced diabetes.

## 4. Materials and Methods

### 4.1. Animals, Induction of Experimental Diabetes, and Sampling

The study was performed on 10 cross-bred (Polish Large White × Polish Landrace) female pigs with initial body weights of approximately 25 kg. The animals were kept in pens under natural light conditions, fed with a commercial grower diet, and had free access to water. Gilts were randomly divided into 2 equal groups, control and diabetic.

After an adaptation period of 7 days, diabetes was induced by the intravenous administration of streptozotocin (Sigma-Aldrich, St. Louise, MO, USA) at a dose of 150 mg/kg of body weight. The pigs were fasted for 18 h before the infusion to avoid vomiting. Streptozotocin was dissolved immediately before used in sodium citrate buffer with pH 4.2 (1 g of streptozotocin per 10 mL of the buffer). The pigs were sedated with ketamine at 10 mg/kg of body weight (Bioketan, Vetoquinol Biowet, Gorzów Wielkopolski, Poland) and the streptozotocin solution was infused for approximately 5 min into the marginal ear vein. Control pigs received citrate buffer. To avoid temporary hypoglycaemia, each animal received 250 mL of 50% glucose solution.

Glucose levels were measured in capillary blood samples collected from the ear every week. The assay was performed using an Accent-200 (Cormay, Warsaw, Poland) biochemical analyser. The results of glucose measurements are presented in Table 1. 

After 6 weeks, the animals were euthanized between 12.00 and 14.00 by overdose of sodium pentobarbital. The pineal glands were immediately removed and cut in the sagittal plane into two equal parts: The right half was frozen at −75 °C for biochemical analysis and the left half was used for in vitro studies in a superfusion culture system. 

Five additional pigs were euthanized as described above to take the pineal glands for control group A in the study in a superfusion culture system.

All experimental procedures on animals were performed in accordance with Polish and European Union laws. They were approved by Local Ethics Committee for Experiments on Animals in Olsztyn, Poland (decision No 45/2013 from 18 September 2013 and decision No 13/2015/DTN from 25 March 2015). The number of animals with streptozotocin-induced diabetes was limited to five due the law regulation on animal protection.

### 4.2. In Vitro Experiment

#### 4.2.1. Culture Medium

Medium 199 containing Earle’s salt and HEPES (Sigma-Aldrich, St. Louis, MO, USA) was prepared from a powder according to the manufacturer’s instructions (pH adjusted to 7.2 with NaOH) and sterilized by filtration. Before use, a sterile solution of ascorbic acid (Sigma-Aldrich, St. Louis, MO, USA) was added to the medium to give a final concentration of 300 mg per 1000 mL.

#### 4.2.2. Superfusion Culture

Explants of pig pineal glands, prepared under sterile conditions, were wrapped with a nylon mesh and placed in the culture chambers. The lower pool of each chamber was connected to three containers with the culture medium. The medium in each container was continuously gassed with a mixture of 5% CO_2_ and 95% O_2._ The source of medium supplying the culture chamber was changed using the 4-way valve. The upper pool of the culture chamber was connected to a multi-channel peristaltic pump, which forced a flow of medium at a rate of 0.2 mL/min. Incubation of explants was performed at 37.5 °C. The medium fractions were collected every 10 min and frozen at −75 °C until assays. 

#### 4.2.3. Experimental Schedule

The experiment was performed on three groups of explants. Group A comprised the pineal explants prepared from normal, untreated pigs; group B—the pineal explants of saline-treated, control pigs; and group C—the pineal explants of streptozotocin-treated, diabetic pigs. After 6 h of incubation in the superfusion culture, the explants from all groups were stimulated with 10 μM NE for 90 min. Then, the explants of groups B and C were treated with 10 µM insulin for 120 min, stimulated with 10 µM NE for 90 min in the presence of insulin (10 µM), and again incubated in the medium with insulin. At the same time, the explants from group A were incubated and stimulated with NE in the medium without insulin. 

### 4.3. Biochemical Studies

#### 4.3.1. Chemicals for HPLC Assays

The following chemicals of the highest purity were purchased from J. T. Baker Chemicals (Center Valley, PA, USA) and used for preparing mobile phases for HPLC: sodium acetate, disodium EDTA, acetic acid, sodium dihydrogen phosphate, citric acid, 1-octanesulfonic acid sodium salt, and phosphoric acid. Methanol and acetonitrile, both of gradient-grade HPLC purity, were provided by Merck Millipore (Billerica, MA, USA). All standards used in HPLC assays, except 5-HTOL, were purchased from Sigma-Aldrich (St. Louis, MO, USA). 5-HTOL was obtained from Santa Cruz Biotechnology (Dallas, TX, USA). Perchloric acid and sodium hydroxide were provided by Merck Millipore (Billerica, MA, USA). A Bradford protein assay kit was purchased from Bio-Rad (Hercules, CA, USA). Ultrapure water (18.2 MΩ, TOC ≤ 5 ppb), freshly prepared using a Milli-Q integral purification system (Merck Millipore, Billerica, MA, USA), was used in all procedures. 

#### 4.3.2. Chemicals for RIA

Anti-melatonin antibody G/S/704-6483 was purchased from Stockgrand Ltd., University of Surrey, Great Britain, and ^3^H-melatonin (87 Ci/mM) was purchased from PerkinElmer (Waltham, MA, USA). All other chemicals were provided by Sigma-Aldrich (St. Louis, MO, USA). 

#### 4.3.3. Sample Preparation for HPLC Assays

The pieces of pineal glands were sonicated in 100 μL of ice-cold 0.1 M perchloric acid using a Vibra-Cell VC 70 ultrasonic processor equipped with a 2-mm probe (Sonics & Materials Inc., Newtown, CT, USA). Each homogenate was incubated for 15 min in an ice-bath, followed by centrifugation at 60,000 *g* (4 °C) for 15 min (Allegra 64R, Coulter Beckman, Indianapolis, IN, USA). The supernatants were carefully transferred into appropriate autosampler vials (La-Pha-Pack Werner Reifferscheidt GmbH, Langerwehe, Germany), and the pellets were frozen at −75 °C for the protein assay. 

The culture medium samples were mixed with 1 M perchloric acid in a proportion of 450:50, incubated for 15 min in an ice-bath, and centrifuged at 60,000× *g* (4 °C) for 15 min (Allegra 64R, Coulter Beckman, Indianapolis, IN, USA). The supernatants were carefully transferred into appropriate autosampler vials (La-Pha-Pack Werner Reifferscheidt GmbH, Langerwehe, Germany). 

#### 4.3.4. Assay of Melatonin Synthesis-Related Indoles

The content of indoles in the pineal gland homogenates was measured using gradient elution HPLC with fluorescence detection according to a previously described and validated procedure [49]. The chromatographic system consisted of an LPG 3400A pump with a built-in degasser, WPS 3000SL autosampler, TCC 3100 column thermostat, and FLD 3400RS fluorescence detector (Dionex, Sunnyvale, CA, USA). A Hypersil GOLD aQ column, with a 3-μm particle size and dimensions of 150 × 4.6 mm (Thermo Scientific, Waltham, MA, USA) kept at 30 °C, and the mobile phase prepared through on-line mixing of methanol and a aqueous solution of 5 mM sodium acetate and 0.01 mM disodium EDTA (pH 4.5) were used to separate the analytes. The initial content of methanol was 10% (*v/v*). Between 9 and 20 min of the separation, the methanol concentration was linearly increased to 30% (*v/v*) and then maintained at a constant level. The flow rate of the mobile phase was 1 mL/min. The injection volume of the standard solutions and samples was 10 μL. The detection was performed at an excitation wavelength of 280 nm and an emission wavelength of 345 nm, at 45 °C. The sensitivity of the detector was changed at 3.80 min of the separation from level 5 to level 1, at 5.00 min of the separation to level 4, at 7 min of the separation to level 6, at 7.75 min of the separation to level 8, and at 23.8 min of the separation to level 7. The chromatograms were analysed using Chromeleon 6.8 software (Dionex, Sunnyvale, CA, USA). 

The limits of quantification (S/N ratio of 10:1 and RSD ≤ 15%) for 5-HTRP, 5-HTOL, NAS, 5-MTAM, 5-MIAA, 5-MTOL, and melatonin were 2.5 pg per injection. The levels of TRP, 5-HT, and 5-HIAA were assayed using a lower sensitivity detector setting, reflecting their high content in the investigated pineal glands, and therefore the limit of quantification for these compounds was 10 pg per injection. The intra-day precision (RSD of peak area) was below 3%, and the inter-day precision was below 4%. 

The same method was used for assay of NAS and melatonin in the culture medium samples, except the injection volume of samples and standards prepared in culture medium was 100 μL. The limits of quantification (S/N ratio of 10:1 and RSD ≤ 15%) for both substances were 10 pg per injection. The intra-day and inter-day precision values (RSD of peak area) were below 4%.

#### 4.3.5. Assay of Catecholamines and Their Metabolites

The contents of catecholamines and their metabolites were measured using HPLC with coulometric detection as described previously [49], using a chromatographic system composed of an LPG 3400M four-channel pump with a built-in degasser (Dionex, Sunnyvale, CA, USA), WPS 3000SL autosampler (Dionex, Sunnyvale, CA, USA) and a CoulArray 5600A electrochemical detector equipped with a four-channel 6210 coulometric cell (ESA Inc., Chelmsford, MA, USA). The standards and samples were injected at 10 µL volumes onto the MG-150 column (3-μm C18, 150 × 3.2 mm, ESA, Inc, Chelmsford, MA, USA) and the separation of analytes was performed (at 25 °C) using a mobile phase consisting of acetonitrile and a buffer containing 90 mM sodium phosphate dihydrate, 50 mM citric acid, 1.7 mM 1-octanesulfonic acid sodium salt, and 50 μM disodium EDTA (pH 3.05 with phosphoric acid), mixed together in a proportion of 5.5:94.5 (*v/v*). The flow rate of the mobile phase was 0.5 mL/min. The potentials applied on consecutive electrodes were as follows: −150, 200, 350, and 450 mV. The data acquisition and integration of chromatograms were performed using CoulArray 3.10 Data Station (ESA Inc., Chelmsford, MA, USA) software. The limits of quantification (10-fold S/N ratio and CV ≤15%) for all measured compounds were below 10 pg per injection. The intra- and inter-day precision (% RSD) values for all analyzed compounds did not exceed 10%.

#### 4.3.6. Protein Assay

The protein content in the pellets obtained after centrifuging the pineal homogenates was measured using a Bradford microplate assay as previously described [49].

#### 4.3.7. Melatonin RIA

Melatonin concentration in the medium samples collected during the superfusion culture was measured by direct RIA according to the previously described and validated procedure [36]. The sensitivity of the assay was 2.5 pg/tube. The intra- and inter-assay coefficients of variation were below 10%.

### 4.4. Statistical Analyses

Data from measurements of melatonin synthesis-related indoles and catecholamines in the pineal tissue were compared using the *t*-test (the in vivo experiment). The data concerning melatonin secretion and NAS release obtained in the superfusion culture were analysed by repeated-measures ANOVA and the LSD test. The analyses were performed using Statistica software (Version 10.0 PL, StatSoft, Tulsa, OK, USA, 2011). A value of *p* ≤ 0.05 was considered as significant. 

## 5. Conclusions

The obtained data show that streptozotocin-induced diabetes significantly affects the sympathetic neurotransmission and metabolism of melatonin synthesis-related indoles in the pig pineal gland. Diabetes results in decreased levels of DOPA, DA, DOPAC, and NE as well as increased levels of VMA. The influence on sympathetic input seems to be one of the pathways leading to changes in pinealocytes as a result of the disease. The most prominent effect of diabetes on pig pinealocytes is a decreased content of 5-HT, which obviously results in diminished levels of 5-HIAA, 5-HTOL, 5-MIAA, 5-MTOL, and 5-MTAM. The synthesis of 5-HT is not affected because there was no change in the level of 5-HTRP; therefore, two mechanisms leading to the decrease in 5-HT content should be considered: increased utilization of 5-HT for NAS synthesis and a defect in storage of 5-HT. The pineal content of NAS is significantly higher in diabetic pigs than in control animals; however, this effect is not reflected in an increased melatonin level. Our in vitro experiment showed no differences in the basal and adrenergic-stimulated release of NAS and melatonin between the pineal glands of diabetic and control pigs. Moreover, the experiment demonstrated the lack of effect of insulin on the release of these indoleamines from the pig pineal gland. 

## Figures and Tables

**Table 1:** Serum glucose levels in pigs of the control and experimental groups.

Sampling Time	Control Group Mean ± SEM (mmol/L)	Experimental Group Mean ± SEM (mmol/L)
Before streptozotocin injection	5.01 ± 0.10	5.030 ± 0.10
1 week after streptozotocin injection	5.08 ± 0.10	17.36 ± 0.38
2 weeks after streptozotocin injection	4.91 ± 0.18	20.72 ± 0.24
3 weeks after streptozotocin injection	5.19 ± 0.06	21.58 ± 0.27
4 weeks after streptozotocin injection	5.31 ± 0.12	20.08 ± 0.09
5 weeks after streptozotocin injection	4.84 ± 0.32	22.26 ± 1.21
6 weeks after streptozotocin injection	5.20 ± 0.10	21.45 ± 1.11

## Abbreviations

TRP	tryptophan
5-HTRP	5-hydroxytryptophan
5-HT	serotonin
NAS	N-acetylserotonin
5-HIAA	5-hydroxyindole acetic acid
5-HTOL	5-hydroxytryptophol
5-MIAA	5-methoxyindole acetic acid
5-MTOL	5-methoxytryptophol
5-MTAM	5-methoxytryptamine
NE	norepinephrine
DA	dopamine
DOPA	3,4-dihydroxyphenylalanine
DOPAC	3,4-dihydroxyphenylacetic acid
VMA	vanillylmandelic acid
HPLC	high pressure liquid chromatography
RIA	radioimmunoassay
